# Factor XIII Measurement and Substitution in Trauma Patients after Admission to an Intensive Care Unit

**DOI:** 10.3390/jcm11144174

**Published:** 2022-07-19

**Authors:** Moritz Katzensteiner, Martin Ponschab, Herbert Schöchl, Daniel Oberladstätter, Johannes Zipperle, Marcin Osuchowski, Christoph J. Schlimp

**Affiliations:** 1 Department of Anaesthesiology and Intensive Care Medicine, AUVA Trauma Center Linz, 4010 Linz, Austria; m.katzensteiner@stud.pmu.ac.at (M.K.); martin.ponschab@auva.at (M.P.); 2 Paracelsus Medical Private University, 5020 Salzburg, Austria; 3 Department of Anaesthesiology and Intensive Care Medicine, AUVA Trauma Center Salzburg, 5010 Salzburg, Austria; herbert.schoechl@auva.at (H.S.); daniel.oberladstaetter@auva.at (D.O.); 4 Ludwig-Boltzmann-Institute Traumatology, The Research Center in Cooperation with AUVA, 1200 Vienna, Austria; johannes.zipperle@trauma.lbg.ac.at (J.Z.); marcin.osuchowski@trauma.lbg.ac.at (M.O.)

**Keywords:** trauma-induced coagulopathy, diagnosis of TIC, bleeding control in major trauma, treatment strategies in severe bleeding trauma patients, coagulation factor XIII, intensive care unit, injury severity score, transfusion requirement, ICU-free days

## Abstract

Trauma patients admitted to an intensive care unit (ICU) may potentially experience a deficiency of coagulation factor thirteen (FXIII). In this retrospective cohort study conducted at a specialized trauma center, ICU patients were studied to determine the dependency of FXIII activity levels on clinical course and substitution with blood and coagulation products. A total of 189 patients with a median injury severity score (ISS) of 25 (16–36, IQR) were included. Abbreviated injury scores for extremities (r = −0.38, *p* < 0.0001) but not ISS (r = −0.03, *p* = 0.45) showed a negative correlation with initial FXIII levels. Patients receiving FXIII concentrate presented with a median initial FXIII level of 54 (48–59)% vs. 88 (74–108)%, *p* < 0.0001 versus controls; they had fewer ICU-free days: 17 (0–22) vs. 22 (16–24), *p* = 0.0001; and received higher amounts of red blood cell units: 5 (2–9) vs. 4 (1–7), *p* < 0.03 before, and 4 (2–7) vs. 1 (0–2), *p* < 0.0001 after FXIII substitution. Matched-pair analyses based on similar initial FXIII levels did not reveal better outcome endpoints in the FXIII-substituted group. The study showed that a low initial FXIII level correlated with the clinical course in this trauma cohort, but a substitution of FXIII did not improve endpoints within the range of the studied FXIII levels. Future prospective studies should investigate the utility of FXIII measurement and lower threshold values of FXIII, which trigger substitution in trauma patients.

## 1. Introduction

Severe traumatic brain injury and serious uncontrolled bleeding remain the major causes of trauma-related mortality [1,2]. Trauma-induced coagulopathy (TIC), a distinct endogenous sequela of hemorrhagic shock and hypoperfusion, can be observed in one-quarter to one-third of all severely injured patients upon hospital admission [3,4,5]. This early endogenous coagulopathy is associated with high transfusion requirements, multi-organ dysfunction syndrome and an almost 4-fold increase in mortality [3]. Fibrinogen has been identified as the first coagulation factor reaching critically low levels in trauma-related blood loss. Moreover, clinical studies revealed a strong association between the severity of shock and low fibrinogen plasma concentrations upon emergency room admission [6]. However, fibrinogen is an acute-phase protein with a strongly upregulated resynthesis rate, resulting in supranormal levels two to three days after trauma [7].

Another contributor to clot strength is coagulation factor XIII (FXIII), which stabilizes a fibrin clot by cross-linking fibrin monomers and incorporating α2-antiplasmin into the clot, thereby protecting the clot from premature lysis. Thus, a decline in FXIII results in poor clot stability and higher susceptibility to fibrinolysis. The low resynthesis rate of FXIII (9–10 days to reach baseline) may predispose severely injured patients to delayed bleeding events in the course of their intensive care unit (ICU) stay despite the high-circulating fibrinogen. Bleeding complications may prolong the length of the ICU stay and increase financial burden [8,9].

We are not aware of studies evaluating the significance of (A) FXIII measurement and (B) FXIII substitution in traumatized patients in the ICU. By performing a retrospective data analysis of trauma ICU patients, we aimed to answer the following questions: (A) Do FXIII levels correlate with injury severity and clinical course? (B) Is there an association between FXIII substitution and clinical outcome?

## 2. Materials and Methods

Ethical approval for the study (committee number 03/2021) was provided by the ethics committee of the AUVA (*Allgemeine Unfall-Versicherungs-Anstalt*) in Vienna. The procedures performed were in accordance with the Declaration of Helsinki from 1975, revised in 2013.

### 2.1. Recruitment of Patient Data

**Figure 1:**
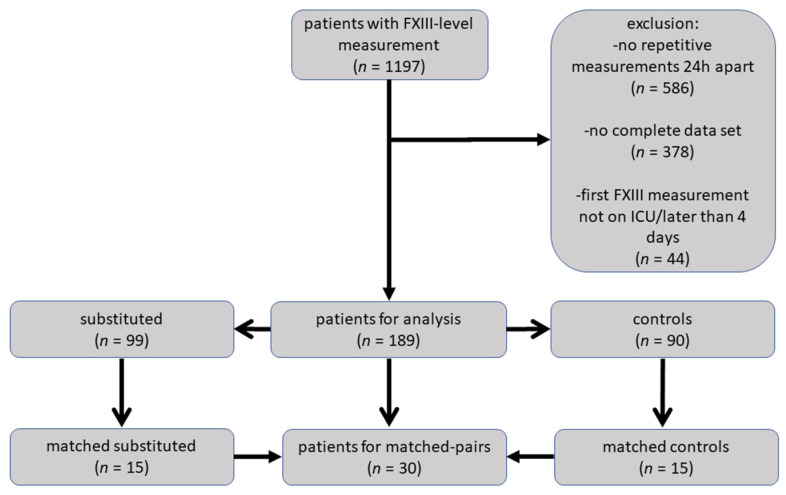
Flowchart depicting patient selection and enrollment according to predefined criteria. All patients were retrospectively recruited for the study. FXIII, coagulation factor XIII; ICU, intensive care unit.

Data were retrospectively collected from the digital patient management system COPRA^®^ (computer organized patient report assistant by COPRA system GmbH in Berlin, Germany) and the hospital patient information database ASTRA^®^ of the Trauma Center Linz. Screening included all patients (regardless of age and history of anticoagulant or antithrombotic therapy) who: (A) were treated in the ICU at the Trauma Center Linz between January 2015 and September 2020, (B) were subjected to an initial FXIII post-admission measurement and (C) the second FXIII measurement approximately 24 h after the first one. Patients with missing data records and those subjected to a FXIII measurement before ICU admission and/or after the fourth post-trauma day were excluded from the study. A decision-making flow chart outlining inclusions/exclusions is displayed in Figure 1.

### 2.2. Data of Patients’ Characteristics

The demographic data of the patients including age, weight, sex, the injury severity score (ISS), the abbreviated injury score (AIS) corresponding to each body section, the length of stay in the ICU, the total number of operations and mortality were collected. ICU-free days, as a more sensitive primary outcome parameter for clinical trials in critically ill patients, were calculated by subtracting the length of stay for the individual patients in days from 30. The ICU-free day value of 0 (zero) reflected either the death of the patient or a longer than 30-day stay in the ICU [10].

### 2.3. Blood Laboratory Analyses

Blood collection, as well as the storage and transportation of blood samples to the central laboratory of the Kepler University Hospital in Linz via pneumatic tubes, was in accordance with the hospital and ICU standards concerning patient safety and hygiene. The FXIII activity level in citrated blood was measured with the Berichrom^®^ FXIII assay (Siemens AG, Berlin/Munich, Germany) with a normal range of 70 to 140%. Base excess (BE, normal range −2.7 to 2.5 mmol/L); hemoglobin (Hb, normal range 12.3 to 17 g/dL); platelet count (Plt, normal range 150 to 400 × 1000/μL); white blood cell count (WBC, normal range 3.9 to 10 × 1000/μL); fibrinogen (normal range 200 to 400 mg/dL); prothrombin time index (PTI, normal range 70–140%); activated partial thromboplastin time (aPTT, normal range 23.0–33.2 s); antithrombin-III activity (ATIII, normal range 75–125%); and C-reactive protein (CRP, normal range < 0.5 mg/dL) were extracted from the hospital databases.

### 2.4. Substituted Blood/Coagulation Factor Products

This data collection included the administration of allogeneic blood products provided by the local blood bank, such as red blood cell (RBC) units, platelet concentrate (PC) units and solvent detergent plasma (SDP) units. Substituted coagulation factor products were FXIII concentrate (Fibrogammin^®^, CSL Behring, Marburg, Germany), fibrinogen concentrate (Haemocomplettan^®^, CSL Behring, Marburg, Germany or Fibryga^®^, Octopharma GmbH, Vienna, Austria), prothrombin complex concentrate (PCC, Beriplex^®^, CSL Behring, Marburg, Germany or Prothromblex^®^, Baxter/Takeda, Vienna, Austria) and antithrombin-III concentrate (Kybernin^®^, CSL Behring, Marburg, Germany).

### 2.5. Definitions of Parameters

The measurement of FXIII and other laboratory parameters occurred at two different time points (TP1 and TP2). TP1 reflects the initial FXIII measurement, while TP2 was measured approximately 24 h after TP1. A surgical intervention was defined as any larger invasive therapeutic intervention disrupting the patients’ body integrity. This included pleural punctions, chest tube placements or surgical/non-surgical tracheotomies as well as recurrent debridements, surgical bandage changes and surgeries per se (performed in and outside of the operating room). The placement of central lines was not considered as surgical intervention.

### 2.6. Statistical Analyses

The distribution of data was assessed using the Kolmogorov–Smirnov test. Given that most datasets were abnormally distributed, the variables are presented as the median, interquartile range (IQR) and minimum-to-maximum range (TR). Data are presented with a violin plot as the median, IQR and TR. Correlations are depicted by a linear graph with the correlation coefficient (r) and *p*-value listed.

In a first step, a Spearman correlation was performed to find any possibly existing dependency of FXIII on the other available determinants. Next, clinically relevant clusters were built and further analyzed with a one-way ANOVA and Tukey’s post hoc test for normally distributed data, or a Kruskal–Wallis and Dunn’s post hoc test for abnormally distributed data.

In the second step, we designated patients without FXIII supplementation as the “control group” and FXIII substituted patients as the “FXIII group”. Intergroup comparison was performed with the non-parametric Mann–Whitney-U test. The Fisher’s exact test was used to analyze contingency data.

The last analytical step was to match patients from both groups (blinded from outcome parameters) using the following criteria: (A) similar initial FXIII level with a maximum difference of 5% (absolute activity) or less; (B) AIS_extremity_ values within the same cluster of 0–2 or 3–5; (C) ICU-free days more than 0 and less than 30. Furthermore, other criteria, when applicable, such as nearest ISS, age, initial RBC and FC units’ administration were considered. A paired *t*-test was used in the matched-pair analysis.

Descriptive and statistical analyses and graphs were generated using GraphPad Prism 9 (GraphPad Software Inc., San Diego, CA, USA). The level of significance was set with a *p*-value of <0.05.

## 3. Results

In the total of 189 retrospectively included patients, we identified two cohorts: (A) patients who did not receive FXIII substitution (*n* = 90, control group) and (B) patients substituted with FXIII concentrate (*n* = 99, FXIII group). Within the 189 patients, we identified 30 subjects that could be matched with regard to the similar initial FXIII level and clinical and demographic data before substitution.

The overall descriptive patient data as well as the number of allogeneic blood transfusions and coagulation factor concentrates given prior to the first measurement (time point TP1) are depicted in Table 1. The first FXIII assessment was performed in a median of 0.5 (0.5–1) days after emergency room admission. A median of 2 (1–4) surgical interventions per patient were performed during their ICU stay. Of note, a single patient with severe burn injuries underwent 74 interventions. The median ICU-free days was 20 (6.5–24). The mortality rate of the investigated cohort was 4.2%.

### 3.1. Correlation Analysis between Determinants and Initial FXIII Level of All Patients

**Figure 2:**
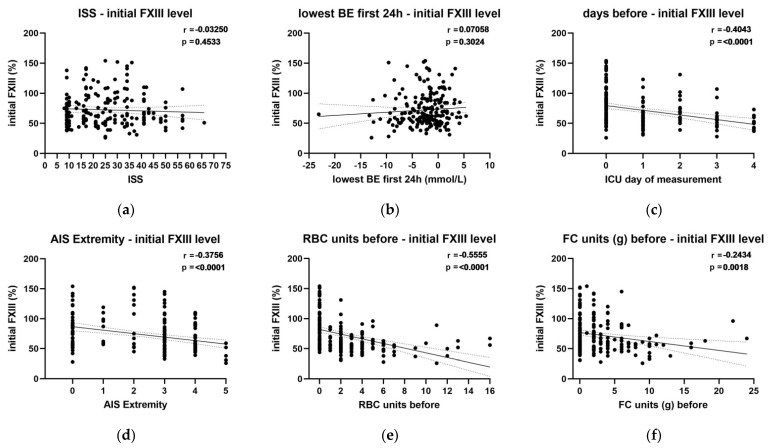
Correlation of highlighted determinants on the initial FXIII level. (**a**): ISS, injury severity score; (**b**): BE, base excess; (**c**): first measurement day post trauma with 0 in the first 24 h, 1 between 24 and 48 h; 2 between 48 and 72 h, 3 between 72 and 96 h, 4 between 96 and 120 h; (**d**): AIS, abbreviated injury score for the extremity region; (**e**): RBC, red blood cell concentrate; (**f**): FC, fibrinogen concentrate.

The complete overall correlation analysis between the determinants and initial FXIII activity is shown in Appendix A Table A1. Notably, no correlation was observed with the ISS or hemorrhagic shock defined by the base excess within the first 24 h after admission (Figure 2a,b). A negative correlation was observed between FXIII and: (A) the time of first sampling (i.e., day of post-trauma measurement; Figure 2c), (B) the severity of extremity trauma (defined by AIS_extremity_; Figure 2d), (C) the amount of transfused RBC units (Figure 2e) and (D) the amount of substituted fibrinogen concentrate (Figure 2f).

### 3.2. Clustered Analysis between Determinants and Initial FXIII Level in All Patients

**Figure 3:**
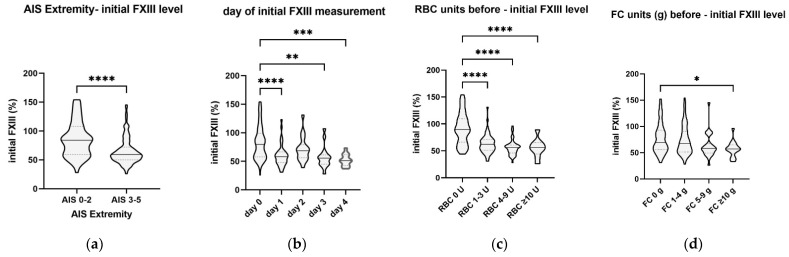
Clustered determinants on the initial FXIII level. (**a**): AIS, abbreviated injury score for the extremity region; (**b**): first measurement day post trauma, with 0 in the first 24 h, 1 between 24 and 48 h; 2 between 48 and 72 h, 3 between 72 and 96 h, 4 between 96 and 120 h; (**c**): RBC, red blood cell concentrate; (**d**): FC, fibrinogen concentrate; level of significance indicates * *p* < 0.05, ** *p* < 0.01, *** *p* < 0.001 and **** *p* < 0.0001; all other comparisons are not significant.

A clustered analysis of the recorded data among the defined cluster groups is displayed in Figure 3. The analysis indicates that AIS_extremity_ ≥ 3 predisposes the patient to low FXIII levels and that the major loss of factor FXIII activity was evident already on the first day after trauma/ICU admission (Figure 3a,b). The need of any RBC transfusion was associated with an FXIII reduction (Figure 3c). In contrast, only high amounts of FC (≥10 g) were associated with lower FXIII activity (Figure 3d).

### 3.3. Initial FXIII Level as a Predictor of Outcome

A significant correlation between an initial FXIII level and the number of surgical interventions, allogeneic blood transfusion and coagulation factor administration was observed. No correlation with ICU-free days was detected (Appendix A Table A1).

### 3.4. Overall Analysis between Control and FXIII Substituted Patients

**Figure 4:**
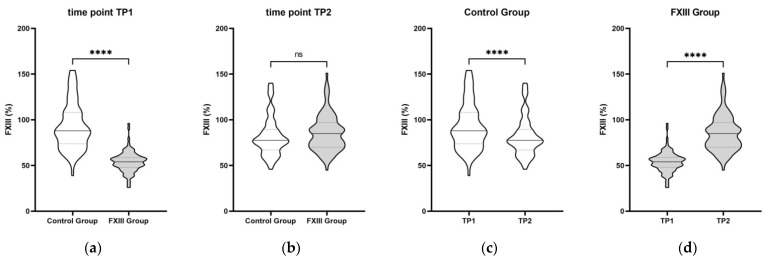
Differences in FXIII levels in the control (*n* = 90) and FXIII (*n* = 99) groups. (**a**) Between groups at TP1; (**b**) between groups at TP2; (**c**) within control group between TP1 and TP2; (**d**) within FXIII group between TP1 and TP2; TP, time point; level of significance indicates **** *p* < 0.0001; ns, not significant.

At TP1, the initial median FXIII level in the control group was 88 (74–108)% versus 54 (48–59)% in the FXIII group (*p* < 0.0001; Figure 4a). After TP1, a median of 1250 (1250–1250) IU of FXIII concentrate was substituted in the FXIII group. At TP2 (approximately 24 h after TP1), the control group had a median FXIII activity level of 77.5% (67–89%), while the FXIII group displayed 85% (70–97%) (*p* = 0.11; Figure 4b). Figure 4c,d display significant intra-group differences between TP1 and TP2.

Notably, PTI, aPTT and fibrinogen were within the normal ranges in the FXIII group (Appendix A, Table A2).

Compared to the control group, patients in the FXIII group were significantly more injured, presented with a lower BE after trauma, had greater organ function impairment (by SOFA score) and displayed an increased need for blood and coagulation products before the initial FXIII measurement and potential substitution (Table 1).

Regarding outcome parameters, the FXIII group showed significantly fewer ICU-free days, more surgical interventions and an increased need for supplementation with blood and coagulation products (Table 1 and Table 2).

### 3.5. Matched-Pair Analysis between Control and FXIII Supplemented Patients

We identified 30 patients (15/group) who could be matched for the similar initial FXIII levels, comparable AIS_extremity_, ISS, BE (within the first 24 h) and clinical course before FXIII substitution (Table 3). In line with a highly significant dependency of the initial FXIII level on the RBC use, a significant blood unit difference of 2 (0–2) vs. 3 (1–4) units (*p* = 0.047) was shown between matched controls and substituted patients. Other initial blood and coagulation products were not significantly different. Similarly, no differences in surgical interventions and coagulation factor use were found. Significant differences in RBC administration and ICU-free days could be detected.

**Figure 5:**
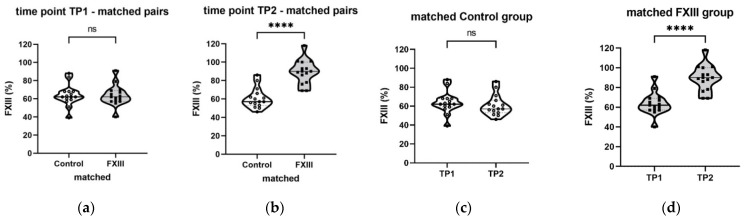
Differences in FXIII levels in the matched control (*n* = 15) and matched FXIII (*n* = 15) groups. (**a**) Between matched groups at TP1; (**b**) between matched groups at TP2; (**c**) within matched control group between TP1 and TP2; (**d**) within matched FXIII group between TP1 and TP2; TP, time point; level of significance indicates **** *p* < 0.0001; ns, not significant.

There was a substantial increase in the FXIII level in the FXIII group between TP1 and TP2 compared to the control patients (Figure 5). Of note, the white blood cell count was significantly higher at TP1, whereas the ATIII level was significantly lower at TP2 in the FXIII group (Appendix A, Table A3).

## 4. Discussion

Our study revealed that the initial FXIII level measured in the ICU was correlated with the extent of tissue trauma (reflected by the AIS_extremity_) as well as with the need for RBC transfusion. Surprisingly, the FXIII level did not correlate with the overall ISS and shock (reflected by the base excess). Furthermore, FXIII-substituted patients presented with higher transfusion needs and a lower initial FXIII level. However, an administration of FXIII concentrate increased the FXIII level to that of the non-substituted control patients. A matched-pair analysis between patient cohorts featuring the same initial FXIII level of median 62% and similar injury pattern did not demonstrate an advantage of FXIII substitution for any assessed outcome parameter.

Until now, no randomized controlled studies investigated the impact of FXIII activity measurement and the consecutive administration of FXIII concentrate on the blood loss and transfusion requirements in severely injured patients. Blood loss and tissue trauma in severely wounded patients result in an activation of profibrinolytic pathways, glycocalyx damage, hypoperfusion-related acidosis and the dilution of the remaining coagulation factors mostly due to a crystalloid fluid-replacement therapy [5]. The above and an insufficient cross-linking of fibrin monomers due to an FXIII deficiency result in poor clot quality and higher susceptibility to clot lysis [11,12]. However, the impact of a low FXIII level on the blood loss in trauma is poorly understood.

In the current study, the overall injury severity calculated using the ISS was not associated with a low FXIII level. However, in the subgroup of extremity trauma (assessed by AIS_extremity_), a significant correlation between the extent of injury and FXIII level was detected. This finding potentially reflects the presence of more pronounced tissue trauma, compared to other AIS categories, with a higher extent of FXIII consumption. Johansson et al. also reported significantly lower FXIII levels in patients with trauma-induced coagulopathy (TIC) compared to non-TIC patients [13]. The ISS, pH and IL-6 in the TIC cohort was significantly worse compared to controls. However, in contrast to our findings, Johansson et al. did not assess the difference based on AIS_extremity_.

Surprisingly, no correlation whatsoever was observed between FXIII level and the shock severity. Intuitively, a high shock burden due to severe bleeding should impact the FXIII level. The above finding is somewhat confounded by the fact that the FXIII level was strongly negatively correlated with the amount of RBC units transfused prior to the first FXIII evaluation.

Controversy remains as to what particular threshold should be used as a decision-making guide for FXIII administration. The last perioperative bleeding guideline from the European Society of Anaesthesiology and Intensive Care suggests an FXIII administration when its level decreases below 30% activity; the suggestion is graded as a weak 2C expert opinion [14]. The latest 2020 German cross-section hemotherapy guidelines recommend an overall target value for FXIII treatment of >50% in patients with a low FXIII level and undergoing a major surgery [15]. However, the latter guidelines do not specifically address FXIII treatment in trauma patients. Moreover, the previous version of the 2014 German cross-section hemotherapy guidelines (we began data collection in 2015) failed to include any recommendation regarding FXIII. A joint, trauma-specific European guideline on the management of major bleeding and coagulopathy following trauma has not yet identified any specific FXIII threshold value at which FXIII should be administered [16]. A recent review from a trauma expert group suggests that a cut-off for FXIII activity of ~60–70% is appropriate to diagnose an acquired FXIII deficiency [12].

Observational prospective studies reported that low FXIII is associated with higher perioperative bleeding risks [17,18]. However, these findings were primarily related to elective surgical procedures and cardiac surgery. For trauma patients, the evidence is by far less clear. Data from animal studies revealed that high-dose FXIII administration in a rat trauma shock model resulted in a reduced bleeding frequency and increased survival [19]. Our current study demonstrated that trauma patients treated with FXIII concentrates received a significantly higher volume of allogeneic blood and a higher amount of coagulation factor concentrates prior to treatment compared to the control group (without FXIII substitution). This finding reflects a higher bleeding tendency in the FXIII-substituted patients compared to the trauma patients, in whom FXIII was measured but not substituted.

FXIII augmentation is difficult to establish with plasma transfusion alone. Given that FXIII in plasma does not typically exceed 1.2 U/mL, it is not possible to adequately treat an FXIII deficit with plasma transfusion alone [20]. Some European trauma centers, which established coagulation-factor-based treatment algorithms, included FXIII therapy following a transfusion of a certain amount of RBCs [21,22]. Those studies reported significantly lower transfusion rates in patients supplemented with coagulation factors compared to non-supplemented controls. However, the exact impact of FXIII concentrate administration is difficult to determine from those investigations.

Several studies highlighted a correlation between the length of stay in the ICU and the amount of RBC transfusion and identified the number of transfused RBC units as an independent risk factor for a prolonged ICU stay and poor outcomes [23]. Thus, early bleeding control is essential for improving outcomes [24]. Duque et al. recently demonstrated that an FXIII level <35% is associated with an increase in major bleeding events as well as prolonged ICU stays [25]. However, as the FXIII group lost more blood, both prior to and after FXIII administration (compared to non-substituted patients), it is unlikely that FXIII administration affected bleeding control.

We focused on FXIII in trauma patients given the scarce information regarding FXIII dynamics and its influence on outcomes in that particular patient group. Apart from the prospective RETIC study (100 enrolled patients; 38 patients received FXIII concentrate) [21], our study is, to the best of our knowledge, the largest trauma patient data collection (189 subjects) with at least two consecutive FXIII measurements in the ICU and featuring matched patients with and without FXIII treatment. The matched patients had similar injury patterns, shock characteristics, basic demographic and clinical data and an equal median FXIII level of 62% at TP1. Expectedly, the FXIII level increased after FXIII administration reaching a normal level of approximately 90% activity at TP2. Interestingly, the FXIII level slightly but significantly decreased to approximately 57% in the control group at TP2. We speculate that despite similar initial FXIII levels (and other clinical characteristics influencing the decision making regarding FXIII supplementation), the actual administration was based on clinical judgement. This judgement was primarily influenced by the higher transfusion needs prior to supplementation and/or the anticipation of higher transfusion requirements (within the following hours) with the concurrent deterioration of FXIII. Based on the ICU-free day calculation, substituted patients in the matched cohort remained significantly longer (by 2 days) in the ICU. It can be hypothesized that without FXIII augmentation, the bleeding tendency would have been even higher and might have further prolonged the ICU stay. Conversely, it is plausible that FXIII administration had no clinically relevant impact upon the outcome parameters assessed in the study.

### 4.1. Limitations

There are several limitations to our study. This was a retrospective FXIII analysis in severely injured trauma patients admitted to an ICU. The time point for initial FXIII measurement was not pre-defined based on any specific FXIII-related data but selected subjectively. To create a homogenous patient cohort for retrospective analysis, we intentionally excluded patients with initial FXIII measurements in the emergency room (i.e., pre-ICU) and only included those with FXIII measurements in the ICU on days 0–4.

The number of matched patients (*n* = 30) was low. This was due to the restrictive matching criteria leading in turn to the median FXIII value of 62% to enable appropriate matching overlaps. Notably, within the study period of 5 years, we could only identify 31 patients with FXIII values < 50%, of whom all but one were substituted with FXIII.

Finally, FXIII was not administered according to an applied standard operating procedure but at the discretion of an attending intensivist.

### 4.2. Future Perspectives

Due to difficulties in a retrospective evaluation of clinical outcomes, prospective randomized clinical trials examining the effect of both FXIII measurement and FXIII substitution in trauma patients are highly warranted. Establishing a decision-making threshold for FXIII substitution is of special interest. However, a focus should be put not only on the FXIII dynamics but more importantly on clinical parameters such as diffuse bleeding.

## 5. Conclusions

We demonstrated that a low initial FXIII level correlates with the clinical course of trauma patients. A high degree of extremity injury as well as the need for blood transfusion rendered the patients prone to a low FXIII level. We also observed that FXIII concentrate administration increased circulating FXIII to that of the control patients. However, we failed to show that FXIII administration reduced bleeding tendency and/or beneficially affected any clinically relevant outcome endpoints. Moreover, we observed that patients in the FXIII-substituted group experienced a prolonged ICU stay and were subjected to a more aggressive substitution with RBCs and fibrinogen. This effect was also observed in the matched-pair analysis. To establish whether FXIII substitution has an outcome-improving potential, lower (than currently tested) threshold values of FXIII, which trigger substitution, need to be tested in prospective randomized clinical trials.

## Figures and Tables

**Table 1:** Demographic and clinical data and allogeneic blood transfusion and coagulation factor concentrate administration prior to the initial FXIII level measurement of all included patients.

Variables	All Patients	Control Group	FXIII Group	*p*-Value
number of patients	189	90	99	
age, years	57 (42–79) (13–92)	56.5 (38–79.3) (13–92)	58 (43–79) (15–92)	0.95
male, *n* (%)	120 (63.5%)	56 (62.2%)	64 (64.6%)	0.73
weight, kg	74.7 (65–87.3) (41–168)	74.7(65–80.8) (49.5–168)	74.3(64.8–91) (41–121.1)	0.72
ISS	25 (16–36) (8–66)	22 (13–34) (8–57)	27 (17–41) (9–66)	**0.01**
AIS head/neck	0 (0–3) (0–5)	1 (0–3) (0–5)	0 (0–3) (0–5)	0.09
AIS face	0 (0–0) (0–4)	0 (0–1) (0–4)	0 (0–0) (0–4)	**0.02**
AIS thorax	0 (0–3) (0–5)	0 (0–3) (0–4)	1 (0–4) (0–5)	0.08
AIS abdomen	0 (0–3) (0–5)	0 (0–3) (0–5)	0 (0–4) (0–5)	0.52
AIS extremity	3 (0.25–4) (0–5)	3 (0–3) (0–4)	3 (3–4) (0–5)	**<0.0001**
AIS external	1 (0–1) (0–5)	1(0–1) (0–4)	1 (0–2) (0–5)	0.42
BE, mmol/L	−2 (−4.2–0.2) (−23.1–5.4)	−1.6 (−3.4–0.6) (−12.6–4.2)	−2.5(−5.5–−0.2) (−23.1–5.4)	**0.02**
SOFA score	4 (2–8) (0–16)	4 (1–7) (0–14)	5 (2–9) (0–16)	**0.03**
day of FXIII measurement ^a^	0.5 (0.5–1) (0.5–4)	0.5 (0.5–1) (0.5–4)	1 (0.5–2) (0.5–4)	**<0.0001**
ICU-free days	20 (6.5–24) (0–28)	22 (16–24) (0–28)	17 (0–22) (0–28)	**0.0001**
surgical interventions	2 (1–4) (0–74)	2 (1–3) (0–9)	3 (2–5) (0–74)	**<0.0001**
mortality, *n* (%)	8 (4.2%)	3 (3.3%)	5 (5.1%)	0.72
**allogeneic blood products**				
RBC, units, pre TP1	4 (2–8) (0–16)	4 (1–7) (0–14)	5 (2–9) (0–16)	**0.03**
PC, units, pre TP1	0 (0–0) (0–3)	0 (0–0) (0–3)	0 (0–0) (0–2)	**0.004**
SDP, units, pre TP1	0 (0–0) (0–7)	0 (0–0) (0–6)	0 (0–0) (0–7)	0.34
**factor concentrates**				
FC, g, pre TP1	2 (0–4) (0–24)	0 (0–2) (0–11)	3 (0–7) (0–24)	**<0.0001**
PCC, IU, pre TP1	0 (0–0) (0–5400)	0 (0–0) (0–2400)	0 (0–500) (0–5400)	**0.02**
ATIII, IU, pre TP1	0 (0–0) (0–4500)	0 (0–0) (0–1000)	0 (0–0) (0–4500)	**0.0004**

ISS, injury severity score; AIS, abbreviated injury score; BE, lowest base excess in first 24 h; SOFA, sequential organ failure assessment; ^a^ day of first FXIII level measurement, with 0 in the first 24 h, 1 between 24 and 48 h; 2 between 48 and 72 h, 3 between 72 and 96 h, 4 between 96 and 120 h; ICU-free days, 30 minus number of days stayed in the intensive care unit with a stay of 30 days or death at any time resulting in 0; TP1, time point of first FXIII measurement; RBC, red blood cell concentrate; PC, platelet concentrate; SDP, solvent detergent plasma; FC, fibrinogen concentrate; PCC, prothrombin complex concentrate; ATIII, antithrombin-III concentrate; IU, international units; data are presented either with median, (interquartile range), (total range); or for sex and mortality with absolute numbers and percentage, *n* (%); *p*-value refers to the difference between control and FXIII groups. Bold format indicate the significant difference.

**Table 2:** Use of allogeneic blood products and coagulation factor concentrates after initial FXIII level measurement.

Variables	Control Group (*n* = 90)	FXIII Group (*n* = 99)	*p*-Value
**FXIII concentrate**			
FXIII conc., IU, 24 h post subst. ^a^	0 (0–0) (0–0)	1250 (1250–1250) (1250–3750)	**<0.0001**
FXIII conc., IU, total post subst.	0 (0–0) (0–0)	1250 (1250–1250) (1250–23,750)	**<0.0001**
**allogeneic blood products**			
RBC, units, 24 h post subst.	0 (0–0) (0–0)	1 (0–3) (0–20)	**<0.0001**
RBC, units, total post subst.	1 (0–2) (0–12)	4 (2–7) (0–100)	**<0.0001**
PC, units, 24 h post subst.	0 (0–0) (0–0)	0 (0–0) (0–5)	**<0.0001**
PC, units, total post subst.	0 (0–0) (0–6)	0 (0–0) (0–5)	**0.005**
SDP, units, total post subst.	0 (0–0) (0–0)	0 (0–0) (0–13)	**0.02**
**factor concentrates**			
FC, g, 24 h post subst.	0 (0–0) (0–9)	0 (0–0) (0–0)	**<0.0001**
FC, g, total post subst.	0 (0–0) (0–7)	0 (0–2) (0–24)	**0.01**
PCC, IU, total post subst.	0 (0–0) (0–1800)	0 (0–0) (0–4200)	0.16

^a^ subst., substitution with FXIII concentrate in the FXIII group; for controls, subst. refers to the first measurement without substitution. When available, some outcome variables are presented for the first 24 h after measurement of FXIII levels/substitution of FXIII as well as with the total amount of substituted products after measurement/substitution. RBC, red blood cell concentrate; PC, platelet concentrate; SDP, solvent detergent plasma; FC, fibrinogen concentrate; PCC, prothrombin complex concentrate; IU, international units; data are presented with median, (interquartile range), (total range).

**Table 3:** Data of the matched-pair analysis.

Variables	Matched Control Group (*n* = 15)	Matched FXIII Group (*n* = 15)	*p*-Value
initial FXIII, %	62 (59–68) (39–88)	62 (57–69) (40–91)	0.86
ISS	16 (10–36) (9–43)	17 (10–32) (9–41)	0.49
AIS extremity	3 (1–3) (0–4)	3 (0–3) (0–4)	0.19
age, years	74 (37–80) (21–90)	71 (56–83) (46–89)	0.23
BE, mmol/L	−1.4 (−2.2–1.4) (−5.3–3.6)	−2.7 (−5.5–0.9) (−8.2–5.4)	0.19
day of FXIII measurement ^a^	1 (0–1) (0–4)	1 (0–2) (0–4)	0.21
**allogeneic blood products pre TP1**			
RBC, units, pre TP1	2 (0–2) (0–4)	3 (1–4) (0–6)	**0.047**
PC, units, pre TP1	0 (0–0) (0–0)	0 (0–0) (0–0)	-
SDP, units, pre TP1	0 (0–0) (0–0)	0 (0–0) (0–0)	-
**factor concentrates pre TP1**			
FC, g, pre TP1	0 (0–3) (0–4)	1 (0–4) (0–10)	0.25
PCC, IU, pre TP1	0 (0–0) (0–600)	0 (0–0) (0–1800)	0.55
ATIII, IU, pre TP1	0 (0–0) (0–0)	0 (0–0) (0–1000)	0.33
**allogeneic blood product post subst.**			
RBC units 24 h post subst. ^b^	0 (0–0) (0–0)	1 (0–2) (0–4)	**0.005**
PC units 24 h post subst.	0 (0–0) (0–0)	0 (0–0) (0–0)	-
RBC units total post subst.	0 (0–1) (0–8)	3 (1–5) (0–14)	**0.03**
PC units total post subst.	0 (0–0) (0,1)	0 (0–0) (0–3)	0.58
SDP units total post subst.	0 (0–0) (0–0)	0 (0–0) (0–0)	-
**factor concentrates post subst.**			
FC (g) 24 h post subst.	0 (0–0) (0–0)	0 (0–0) (0–0)	-
FC (g) total post subst.	0 (0–0) (0–7)	2 (0–0) (0–10)	0.75
PCC (IU) total post subst.	0 (0–0) (0–1200)	0 (0–0) (0–1200)	>0.99
ATIII (IU) total post subst.	0 (0–0) (0–1000)	0 (0–0) (0–3000)	0.28
**other variables**			
ICU-free days	23 (22–25) (16–28)	21 (19–23) (2–26)	**0.04**
surgical interventions	1 (1–2) (0–5)	2 (1–2) (0–4)	0.51

FXIII, coagulation factor thirteen; TP1, first measurement of FXIII; ISS, injury severity score; AIS, abbreviated injury score; BE, lowest base excess in first 24 h; ^a^ day of first FXIII level measurement, with 0 in the first 24 h, 1 between 24 and 48 h; 2 between 48 and 72 h, 3 between 72 and 96 h, 4 between 96 and 120 h; RBC, red blood cell concentrate; PC, platelet concentrate; SDP, solvent detergent plasma; FC, fibrinogen concentrate; PCC, prothrombin complex concentrate; ATIII, antithrombin-III concentrate; IU, international units; ^b^ subst., substitution with FXIII supplements in the FXIII group; for controls, subst. refers to the first measurement without substitution; ICU-free days, 30 minus number of days stayed in the intensive care unit with a stay of 30 days or death at any time resulting in 0; data are presented with median, (interquartile range), (total range); *p*-value, refers to the difference between matched control and FXIII groups.

## Data Availability

The data presented in this study are available on request from the authors.

## Appendix A

**Table A1:** Correlations between determinants and the initial FXIII level.

Determinant Correlated with Initial FXIII Level	Spearman r (95% CI)	*p*-Value
**predisposing variables**		
age, years	−0.08 (−0.23–0.06)	0.25
weight, kg	0.02 (−0.15–0.18)	0.81
ISS	−0.03 (−0.18–0.12)	0.66
AIS head/neck	0.24 (0.09–0.37)	**0.001**
AIS face	0.18 (0.03–0.32)	**0.02**
AIS thorax	0.01 (−0.14–0.16)	0.88
AIS abdomen	−0.03 (−0.18–0.12)	0.69
AIS extremity	−0.38 (−0.5–−0.24)	**<0.0001**
AIS external	0.01 (−0.14–0.16)	0.90
BE, mmol/L	0.07 (−0.08–0.22)	0.33
day measured ^a^	−0.40 (−0.52–−0.27)	**<0.0001**
SOFA	−0.09 (−0.25–0.07)	0.25
allogeneic blood products		
RBC, units, pre TP1	−0.56 (−0.65–−0.4)	**<0.0001**
PC, units, pre TP1	−0.18 (−0.32–−0.03)	**0.01**
SDP, units, pre TP1	−0.03 (−0.17–0.12)	0.72
coagulation factor concentrates		
FC, g, pre TP1	−0.24 (−0.38–−0.1)	**0.001**
PCC, IU, pre TP1	−0.11 (−0.25–0.04)	0.14
ATIII, IU, pre TP1	−0.23 (−0.37–−0.09)	**0.001**
**outcome variables**		
surgical interventions, *n*	−0.24 (−0.37–−0.1)	**0.001**
ICU-free days	0.12 (−0.03–0.3)	0.10
allogeneic blood products		
RBC, units, 24 h post subst. ^b^	−0.50 (−0.6–−0.38)	**<0.0001**
PC, units, 24 h post subst.	−0.23 (−0.36–−0.08)	**0.002**
RBC, units, total post subst.	−0.39 (−0.5–−0.25)	**0.0001**
PC, units, total post subst.	−0.25 (−0.38–−0.1)	**0.001**
SDP, units, total post subst.	−0.15 (−0.29–−0.01)	**0.04**
coagulation factor concentrates		
FC, g, 24 h post subst.	−0.18 (−0.32–−0.03)	**0.02**
FC, g, total post subst.	−0.12 (0.27–0.02)	0.09
PCC, IU, total post subst.	−0.03 (−0.17–0.12)	0.70
ATIII, IU, total post subst.	−0.25 (−0.38–−0.11)	**0.001**

FXIII, coagulation factor XIII; CI, confidence interval; ISS, injury severity score AIS, abbreviated injury score; BE, base excess; SOFA, sequential organ failure assessment; RBC, red blood cell concentrate; PC, platelet concentrate; SDP, solvent detergent plasma; FC, fibrinogen concentrate; PCC, prothrombin complex concentrate; ATIII, antithrombin-III concentrate; ICU-free days, 30 minus number of days stayed at the intensive care unit, with death or longer stays resulting in 0; ^a^ day of first FXIII level measurement, with 0 in the first 24 h, 1 between 24 and 48 h; 2 between 48 and 72 h, 3 between 72 and 96 h, 4 between 96 and 120 h; ^b^ subst., substitution with FXIII supplements in the FXIII group; for controls, subst. refers to the first measurement without substitution; TP1, time point of initial FXIII measurement.

**Table A2:** Laboratory parameters of all patients (*n* = 189) at the two consecutive time points.

Time Point	Parameter	Overall Control Group (*n* = 90)	Overall FXIII Group (*n* = 99)	*p*-Value
TP1	FXIII level, %	88 (73.8–108.3) (39–154)	54 (48–59) (26–96)	**<0.0001**
TP2	FXIII level, %	77.5 (67–89.3) (46–140)	85 (70–97) (45–151)	0.11
*p*-value		**<0.0001**	**<0.0001**	
TP1	Hb, g/dL	9.5 (8.6–11.5) (6.7–16.6)	8.4 (7.9–9.1) (6.2–12.9)	**<0.0001**
TP2	Hb, g/dL	9.2 (8.4–10.2) (6.7–14.3)	8.3 (7.8–9.1) (6.3–11.5)	**<0.0001**
*p*-value		**0.0015**	0.1	
TP1	Plt, ×1000/µL	165 (129–217.3) (57–664)	120 (90–150) (48–471)	**<0.0001**
TP2	Plt, ×1000/µL	149 (113.5–195.5) (40–667)	115 (88–144.3) (40–504)	**<0.0001**
*p*-value		**<0.0001**	0.09	
TP1	WBC, ×1000/µL	9.7 (7.7–13.5) (3.9–29.3)	8.9 (7–12.3) (3.4–23.3)	0.07
TP2	WBC, ×1000/µL	8.0 (6.2–11.5) (3–33.9)	8.3 (6.2–10.3) (2.3–19)	0.87
*p*-value		**<0.0001**	**0.0035**	
TP1	Fibrinogen, mg/dL	298 (232–408) (60–899)	318 (227–441) (124–900)	0.75
TP2	Fibrinogen, mg/dL	392 (310.8–482.5) (106–900)	393 (291–507) (153–900)	0.66
*p*-value		**<0.0001**	**<0.0001**	
TP1	PTI, %	85 (72.5–91.3) (25–120)	75 (64–92) (31–139)	0.05
TP2	PTI, %	85 (77–97.8) (12–120)	85 (69–98) (38–135)	0.41
*p*-value		**0.044**	**<0.0001**	
TP1	aPTT, sec	26.2 (24–28.9) (17.6–58.9)	29.6 (26.2–36.1) (19.7–160)	**<0.0001**
TP2	aPTT, sec	27.4 (25.3–29.8) (17.2–67.9)	29.0 (25.6–33.7) (21.2–68.5)	**0.02**
*p*-value		**0.0009**	0.13	
TP1	ATIII level, %	84 (72.5–94.5) (40–145)	63 (54–80) (22–119)	**<0.0001**
TP2	ATIII level, %	84 (76–94) (38–123)	70 (59–82.5) (28–115)	**<0.0001**
*p*-value		0.57	**<0.0001**	
TP1	CRP, mg/dL	2.6 (0.6–7.9) (0.1–31.9)	7.5 (3–12.4) (0.1–39.8)	**<0.0001**
TP2	CRP, mg/dL	8.8 (5.5–14.9) (0.1–36)	12.0 (6.8–17.3) (1.3–35.6)	**0.02**
*p*-value		**<0.0001**	**<0.0001**	

TP1, initial measurement of FXIII and other laboratory parameters; TP2, second measurement of the same parameters approx. 24 h later; FXIII, coagulation factor thirteen activity; Hb, hemoglobin; Plt, platelet count; WBC, white blood cell count; PTI, prothrombin time index; aPTT, activated partial thromboplastin time; ATIII, antithrombin-III activity; CRP, C-reactive protein; data are presented with median, (interquartile range), (total range); *p*-value on the right side of table refers to the difference between control and FXIII group (Mann–Whitney-U test); *p*-value below each time point pair refers to the difference between TP1 and TP2 within each group (paired *t*-test).

**Table A3:** Laboratory parameters of 30 patients in the matched cohorts at the two consecutive time points.

Time Point	Parameter	Matched Control Group (*n* = 15)	Matched FXIII Group (*n* = 15)	*p*-Value
TP1	FXIII level, %	62 (59–68) (39–88)	62 (57–69) (40–91)	0.86
TP2	FXIII level, %	57 (53–66) (46–86)	90 (78–100) (69–118)	**<0.0001**
*p*-value		0.25	**<0.0001**	
TP1	Hb, g/dL	9.1 (8.5–9.5) (7.5–12.5)	8.7 (8.2–9.5) (7.5–10.5)	0.35
TP2	Hb, g/dL	8.5 (8.3–9.8) (6.7–12.4)	8.7 (8.3–9.1) (7.7–9.6)	0.4
*p*-value		0.79	0.72	
TP1	Plt, ×1000/µL	117 (91–166) (57–213)	148 (106–181) (70–471)	0.16
TP2	Plt, ×1000/µL	124 (80–165) (48–283)	135 (100–189) (78–504)	0.37
*p*-value		0.52	0.69	
TP1	WBC, ×1000/µL	7.5 (6–8.4) (3.9–15.7)	10.9 (6.1–13.1) (3.4–17.5)	**0.04**
TP2	WBC, ×1000/µL	7.1 (3.9–9) (3–12.3)	9.4 (5.8–10.8) (4.3–17.3)	0.05
*p*-value		**0.02**	0.28	
TP1	Fibrinogen, mg/dL	331 (249–446) (144–659)	345 (232–489) (167–684)	0.92
TP2	Fibrinogen, mg/dL	460 (304–508) (133–632)	390.5 (310.5–502.8) (217–711)	0.92
*p*-value		**0.03**	**<0.01**	
TP1	PTI, %	83 (74–94) (53–118)	80 (70–100) (67–139)	0.73
TP2	PTI, %	92 (77–106) (64–119)	88.5 (79.5–98.8) (62–135)	0.91
*p*-value		**0.04**	0.12	
TP1	aPTT, sec	28.1 (25.8–31.9) (23–34.5)	27.1 (24–29.6) (21.9–59.7)	0.83
TP2	aPTT, sec	27.4 (25.5–29.8) (23–37.2)	25.7 (24.3–30.4) (22.2–55)	0.78
*p*-value		0.32	0.29	
TP1	ATIII level, %	79 (66–92) 45–118)	72 (55–87) (45–119)	0.38
TP2	ATIII level, %	81 (69–100) (38–123)	68 (54.8–88.5) (43–114)	**0.04**
*p*-value		0.09	0.78	
TP1	CRP, mg/dL	6.9 (1–9.5) (0.1–16.6)	10 (3.7–14.7) (0.5–20.5)	0.16
TP2	CRP, mg/dL	8.5 (5.4–16.2) (0.1–18.6)	12.7 (5.9–16) (3.5–19.8)	0.54
*p*-value		**<0.01**	0.09	

TP1, initial measurement of FXIII and other laboratory parameters; TP2, second measurement of the same parameters approx. 24 h later; FXIII, coagulation factor thirteen activity; Hb, hemoglobin; Plt, platelet count; WBC, white blood cell count; PTI, prothrombin time index; aPTT, activated partial thromboplastin time; ATIII, antithrombin-III activity; CRP, C-reactive protein; data are presented with median, (interquartile range), (total range); *p*-value on the right side of table refers to the difference between the matched pairs of control and FXIII group (paired *t*-test); *p*-value below each time point pair refers to the difference between TP1 and TP2 within each group (paired *t*-test).
